# A specialized fungal parasite (*Massospora cicadina*) hijacks the sexual signals of periodical cicadas (Hemiptera: Cicadidae: *Magicicada*)

**DOI:** 10.1038/s41598-018-19813-0

**Published:** 2018-01-23

**Authors:** John R. Cooley, David C. Marshall, Kathy B. R. Hill

**Affiliations:** 1 College of Integrative Sciences, Wesleyan University, Middletown, CT USA; 2 Dept. of Ecology and Evolutionary Biology, University of Connecticut, Storrs, CT USA

## Abstract

Male periodical cicadas (*Magicicada* spp.) infected with conidiospore-producing (“Stage I”) infections of the entomopathogenic fungus *Massospora cicadina* exhibit precisely timed wing-flick signaling behavior normally seen only in sexually receptive female cicadas. Male wing-flicks attract copulation attempts from conspecific males in the chorus; close contact apparently spreads the infective conidiospores. In contrast, males with “Stage II” infections that produce resting spores that wait for the next cicada generation do not produce female-specific signals. We propose that these complex fungus-induced behavioral changes, which resemble apparently independently derived changes in other cicada-*Massospora* systems, represent a fungus “extended phenotype” that hijacks cicadas, turning them into vehicles for fungus transmission at the expense of the cicadas’ own interests.

## Introduction

Parasites and their hosts cohabit the same body, but they have strongly divergent interests in how to make use of it. Parasites modify hosts to create structures or behaviors that may be thought of as “extended phenotypes” serving parasite, not host, reproductive interests^1–5^. While some behavioral changes may expose hosts to predators and thereby effect transmission of the parasite^6^, not all behavioral changes observed in infected hosts are parasite adaptations; some could be interpreted as incidental disease pathologies or host strategies to counteract infection^2,4,6,7^. For instance, Abbot and Dill^8^ describe how milkweed beetles (*Labidomera clivicollis*) parasitized by mites (*Chrysomelobia labidomerae*) show increased short-term reproductive effort. Since mite infections are spread by contact among beetles, such increases in effort could be part of a mite strategy to increase transmission rates; however, increased reproductive effort could also be a strategy of the beetles to salvage the best of a bad situation^8^. Poulin^2,7^ noted that the case for an adaptive parasite “extended phenotype” is made stronger if host behavioral changes are 1) complex, 2) clearly reproductively functional for the parasite, and 3) independently derived in different host lineages. For example, the novelty and complexity of “Zombie ant” (*Camponotus leonardi*) death positioning behaviors when they are infected with the fungus *Ophiocordyceps unilateralis*^4^ seem to benefit the fungus exclusively, one of many similar examples among arthropod-fungus associations^9–11^.

The entomophthoralean fungus *Massospora cicadina* Peck infects periodical cicadas (*Magicicada spp*.) during their regionally synchronized, roughly month-long adult emergences every 13 or 17 years^12,13^ and is the only known predator or pathogen synchronized to the cicadas’ life cycles^14,15^. Mycosis follows a predictable phenology. Early in a periodical cicada emergence, “Stage I” infected cicadas produce haploid conidiospores capable of infecting other actively chorusing adult cicadas^16–18^. Later in the emergence, “Stage II” cicadas infected by conidiospores produce diploid resting spores that fall to the soil and complete the fungal life cycle by infecting the next generation of cicada nymphs due to emerge 13 or 17 years later^18,19^. Stage I *Massospora* infections are acquired as nymphs emerge from the soil^19^, while Stage II infections are spread among adult cicadas of the same generation.

**Figure 1:**
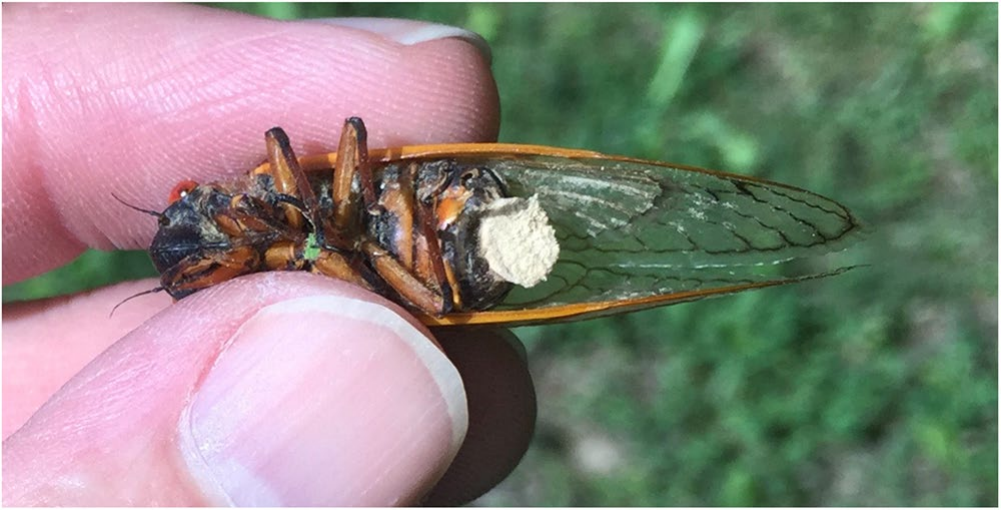
Female *Magicicada septendecim* with *Massospora cicadina* Stage I infection and loss of terminal abdominal segments. Photo credit The Authors.

Entomophthoralean fungi in general are known for subverting host structures and behaviors for their own interests^20^. *Massospora* clearly modifies cicada phenotypes in ways contrary to the cicadas’ interests. Both Stage I and Stage II infections cause distention and loss of the terminal abdominal segments, genitalia included, in both sexes^16–18^; the breached abdomen exposes infective spores and allows their dispersal (Fig. 1). Less clear is whether *Massospora* infections alter cicada *behaviors* in ways that work to the fungus’ advantage. White *et al*.^21^ noted that infected cicadas remain relatively stationary, tend to make shorter flights than those of uninfected cicadas, and tend to drag their abdomens when they walk, leaving trails of spores. R. D. Alexander (pers. comm.) collected data suggesting that cicadas with Stage I infections spend relatively more time than Stage II cicadas walking and leaving trails of conidiospores for other cicadas to encounter, while cicadas with Stage II infections spend relatively more time than Stage I cicadas flying and visibly spewing spores from their damaged abdomens. While it is plausible that these behavioral changes could be part of a fungal extended phenotype, they could also result from the general phenology of a *Magicicada* emergence (e.g. perhaps cicadas change their general activity levels as they senesce) and/or the physical damage caused by the fungus (e.g., perhaps sterilization or other fungal damage accelerates senescence).

While both male and female *Magicicada* are infected by *Massospora*, there is some disagreement as to their relative susceptibility. Cantrall’s^22^ informal survey suggested higher infection rates (unspecified whether Stage I or II) in females than in males, but Speare^16^ noted that infections (unspecified whether Stage I or II) are more prevalent in males. Interestingly, more systematic surveys, such as White and Lloyd^23^, found no difference in Stage I infection rates in males and females but both Lloyd *et al*.^24^ and White and Lloyd^23^ found that males had higher rates of Stage II infections than did females.

Here, we report novel periodical cicada sexual behaviors associated with *Massospora* infections, based on observations we first made while describing and characterizing the sexual behaviors of normal, uninfected individuals^25–27^. These novel behaviors are better explained as manifestations of fungal adaptation than as incidental physiological consequences of infection. In short, *Massospora’s* complex extended phenotype turns it into a unique, specialized sexually transmitted disease to which male periodical cicadas are especially vulnerable.

## Results

**Figure 2:**
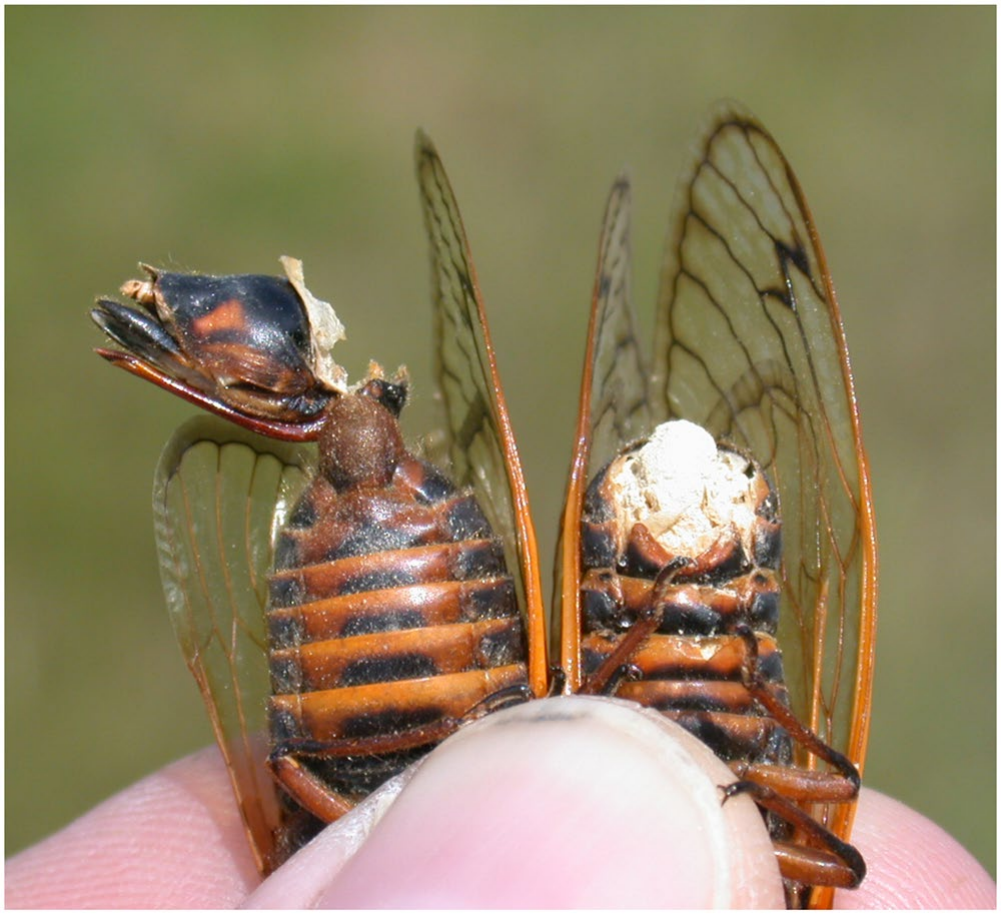
Uninfected male *Magicicada septendecim* (left) with genitalia torn from a female infected by Stage I of *Massospora cicadina* (right). Photo credit The Authors.

*Massospora* -infected cicadas show some normal sexual behaviors despite the physical damage caused by the fungus. Cicadas producing infective spores retain their ability to fly and produce alarm sounds when held, males with both Stage I and Stage II infections sometimes produce calling or courtship sounds, and females with both Stage I and Stage II infections wing-flick as expected^26^ in response to male calls. Even cicadas that have lost the terminal halves of their abdomens behave as if they were sexually responsive and can be seen engaging vigorously in courtship behavior and attempted copulation with other individuals; thus, it is relatively common to find a healthy cicada with its genitalia plunged into the abdominal spore mass of an infected partner^18,23^ or to see healthy cicadas attached to fragments of abdomen or terminalia that have torn free from infected partners during attempted copulation (Fig. 2).

**Figure 3:**
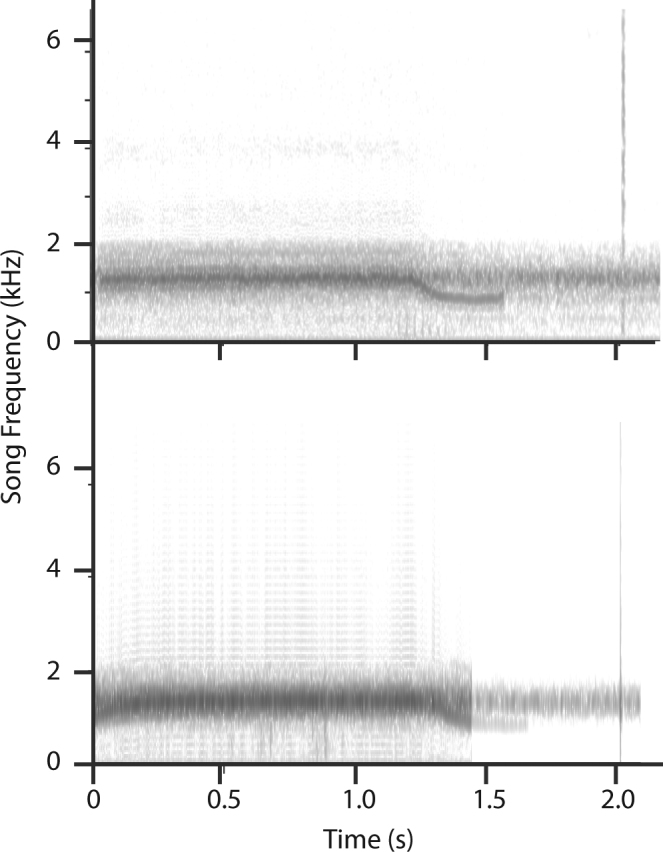
Top panel: Sonogram of male *Magicicada* -decim call and female wing flick response (marked with asterisk), modified from Fig. 4 of Cooley and Marshall^26^. Bottom panel: Sonogram of response of *Magicicada* -decim male with Stage I *Massospora cicadina* infection to an M. -decim call. Male response (marked with asterisk) is a broad-frequency sound similar in timing and acoustical properties to female wing flick signals. In both panels, wing-flick sounds are enhanced and extraneous background noise has been removed for clarity.

In our initial playback experiments, Stage I fungus-infected *Magicicada* responded to playbacks with appropriately timed wing-flicks (Fig. 3), while no normal male or Stage II infected male ever responded. Stage I infected males appear slightly less responsive than mature females (Table 1), although small sample sizes preclude any meaningful statistical analysis. Infected individuals produced the largest number of responses to calls with the species-typical song pitch and showed decreasing responses to altered frequencies, just as normal males did.

**Table 1:** Numbers of positive responses (timed wing flicks) by adult *M. septendecim* to playbacks of normal and frequency-altered *M. septendecim* calling songs.

Song frequency (kHz)	Mature females (32)	Stage I Male (6)	Stage II male (5)	Normal male (8)
2.35	0	0		0
1.91	2	0		0
1.65	12	1		0
1.48	16	2		0
1.39 (unmodified)	22	3	0	0
1.30	20	1		0
1.13	18	0		0
0.956	8	0		0
0.869	1	0		0

In a small series of playbacks to a group of male and female Stage I infected *Magicicada*, both males and females responded positively with wing-flicks in response to playbacks of species-typical male calls, consistent with our observations of free-flying cicadas. Additional playback trials to males of all cicada species demonstrated that Stage I infected males were responsive to conspecific call playbacks while Stage II infected males were not (Table 2).

**Table 2:** Aggregated data from 2002 playbacks to males infected with *Massospora cicadina*. Counts are of individual cicadas that responded vs. cicadas that did not respond to playbacks.

Response	Stage I male	Stage II male
Wing-flick (+)	23	0
No Wing-flick (−)	2	17

Aggregating data from all years (1996–2003), Stage I infected males of various periodical cicada species responded to conspecific song stimuli while no normal male or Stage II infected male ever responded to a conspecific song stimulus (Table 3). These results complement our earlier findings^26^ that normal male cicadas never wing-flick in response to conspecific song stimuli like receptive females do. Our experimental results are bolstered by our many casual observations of wing-flicking by free-flying infected cicadas, none of which have involved Stage II infected males.

**Table 3:** Counts of responding and non-responding cicadas of all species from all years of the study.

Host species	Stage I male responding	Stage II male not responding	Normal male not responding
*M. septendecim*	9	7	23
*M. neotredecim*		11	
*M. tredecim*	23	5	
*M. cassinii*		7	
*M. tredecassini*		1	
*M. septendecula*			
*M. tredecula*	1		
Total	33	31	23

## Discussion

Our results show that changes in the behaviors of adult periodical cicadas caused by *Massospora* infections are more complex than previously realized. Stage I *Massospora* infections, which produce spores that are spread directly to other adult cicadas, cause males to wing-flick in response to the calls of other males, with the same species-specific timing used by sexually receptive *Magicicada* females. These novel wing-flick responses are attractive to normal, uninfected males, who repeatedly attempt to copulate with the diseased males. Stage II infections, which produce spores that are dispersed to the soil to infect the next generation, do not cause males to wing-flick in response to conspecific male song. Although we have not followed the consequences of copulation attempts between infected and uninfected cicadas, we expect that close contact increases the odds of transmission; thus, *Massospora* functions at least partly as a sexually transmitted disease and the novel behaviors of infected males are complex manipulations instigated by the fungus for its own benefit.

The absence of wing-flicking in males with Stage II fungal infections is key for understanding the adaptive nature of fungus-induced behavioral changes. If both fungal stages caused males to express sexual behaviors normally observed in females only, then the changes might be more easily dismissed as mere byproducts of the physical damage suffered by infected cicadas. But the fact that Stage I infected males express both male and female sexual behaviors, while Stage II infected cicadas do not, makes it extremely unlikely that fungal infections cause males to develop a “default” female phenotype, perhaps by causing sterility or by interfering with sexual organ development. Moreover, Stage I infected cicadas exhibit female behaviors in addition to, not instead of, their expected male behaviors. By some as yet unknown means, perhaps involving manipulations of victims’ endocrine or nervous systems, the fungus adds novel behaviors to cicadas’ repertoires.

Along with earlier observations on changing activity patterns of Stage I and II infected cicadas, the sexual behavioral changes documented here appear complex and well-matched to the fungus life cycle—conidiospores are transmitted to coappearing adult cicadas, while resting spores wait for the next generation. Our results also incidentally provide an explanation for earlier reports that males have higher rates of Stage II infections than do females^23,24^: While females are in jeopardy of contracting Stage II infections through sexual contact with Stage I infected males, males are in jeopardy of contracting Stage II infections from sexual contact with Stage I infected individuals of both sexes. The effectiveness of males in dispersing infections is likely compounded by the fact that male periodical cicadas generally attempt to mate more often than do females^28^.

Since fungal infections cause sterility, cicadas should be expected to be under selection to avoid contact with spores; accordingly, females would benefit from an ability to detect early symptoms of fungus infections in potential mates. *Magicicada* have a lengthy and complex courtship sequence^26,29^, which might create opportunities for females to detect infected males, especially if infections alter male songs in detectable ways. However, to date, within-species mate discrimination in *Magicicada* is known to involve only the operation of minimal thresholds met by most actively chorusing conspecifics^28,30^ (but see^31^). Thus, there is no evidence that female *Magicicada* are capable of identifying and avoiding infected males.

Our findings are not unique to the *Magicicada-Massospora* host-parasite system. Cooley^32^ reports similar *Massospora-*induced sexual behavioral changes in the cicada *Okanagana rimosa* (Say), of the subfamily Tibicininae (*Magicicada* are classed in Cicadettinae). *O. rimosa* develop conidiospore-producing infections of *M. levispora* Soper^18,33^ that are similar in appearance to Stage I *M. cicadina* infections of *Magicicada* (Soper *et al*. 1976, White and Lloyd 1983). *Okanagana rimosa* individuals encountering conidiospores produced by Stage I infected cicadas develop Stage II (resting spore producing) *M. levispora* infections that cause no visible external changes (Soper 1963, Soper *et al*. 1976). As in the *Magicicada*-*Massospora cicadina* system, male cicadas with Stage I infections of *M. levispora* exhibit behaviors characteristic of sexually responsive females. *Okanagana* do not use wing-flick signals in the same way as *Magicicada;* sexual receptivity in *Okanagana* is exhibited by tolerance of physical contact by an approaching male, who locates the female visually and approaches after she flies to his vicinity^25^. Normal *O. rimosa* males and unreceptive females have never been observed tolerating physical contact by other cicadas. However, Stage I fungus infected male *O. rimosa* tolerate mounts by courting males, suggesting that this fungus also may manipulate male cicada sexual behaviors to increase its odds of transmission^32^. Other similar examples have been reported; Murphy and Redden^34^ note that crepitating cicadas (*Platypedia putnami*, also in Tibicininae) infected with an unknown *Massospora-*like fungus show intermediate sexual behaviors and are attracted to signaling cicadas of either sex. *Massospora-*like infections are known from several other cicada species (at least one from tribe Cicadettini, unpublished data), and we expect at least some of them to be associated with behavioral changes that render infected individuals sexually attractive. These findings raise the possibility that the general phenomenon of “zombie insects” may also include parasites that specifically hijack host sexual behaviors.

## Methods

In any given area, periodical cicadas emerge predictably once every 13 or 17 years and form dense, multi-species aggregations, or “choruses”^35,36^. Emerging nymphs undergo ecdysis and spend several days maturing as “teneral” adults before joining choruses. Within choruses, males make loud, species-specific acoustical “songs”; receptive females respond by producing wing-flicks with species-specific timing^26^, and males and females mate after a series of stereotypical courtship behaviors^26,35^. The sexual behaviors of the seven currently described periodical cicada species are broadly similar, differing primarily in the details of male calls and the timing of female responses^26^. After separating, females oviposit and do not generally remate following copulations of normal duration^28^; males, on the other hand, continue to pursue prospective mates.

Predicting where to find infected cicadas, especially Stage II infected cicadas, in sufficient numbers is a difficult task since infection rates vary over time and space^23,24^. Thus, we studied the effects of *Massospora* infections opportunistically, in different periodical cicada broods using the periodical cicada species that were available. Over the course of our previous research, we spent many hours observing both caged and free-flying *Magicicada*, some of which were infected with *Massospora*. In 1996, while observing free-flying *Magicicada* in Brood II, we were surprised to observe a Stage I *Massospora*-infected male *M. septendecim* respond to a calling male twice with wing-flick signals; the calling male responded as expected by attempting to copulate with the infected, wing-flicking male. This observation was striking because, in hundreds of hours spent to that point observing *Magicicada* in natural and controlled circumstances, we had never observed normal, uninfected males making wing-flick signals in response to other males’ calls^26^.

Following our initial observations, we conducted playback experiments on caged cicadas using the same methods and song stimuli as in our other studies of periodical cicada signaling^26,37,38^. In our initial playback experiments to normal males, normal females, and Stage I infected males, each playback trial began with a male calling song at normal pitch (1.39 kHz) followed by a series of frequency-altered pitches, concluding with a calling song at normal pitch; if a cicada responded with appropriately timed wing-flick signals^26^ to a minimum of two phrases, it was scored as responding positively. We also tested the responsiveness of Stage II *Massospora*-infected males to playbacks of unaltered calling song.

During subsequent periodical cicada emergences, we conducted playbacks of species-typical songs to fungus-infected cicadas opportunistically, as we worked to further characterize the *Magicicada* mating system during the 1997 emergence of Brood III^26^ and the 2003 emergence of Brood IX, and as we described reproductive character displacement in *M. neotredecim* during the 1998 emergence of Brood XIX^37^, and the 2002 emergence of Brood XXIII^39^. We conducted playback experiments to Stage I and II fungus-infected individual periodical cicadas of various species alongside the uninfected cicadas used in these studies using species-typical calls, the same protocols, and the same criteria for judging responsiveness as before.

### Data availability

All data generated or analysed during this study are included in this published article.
